# 

**Published:** 1890-11

**Authors:** 


### CONTENTS.

Biography of Dr. Joseph Richardson, D.D.S., M D.... 525

COMMUNICATIONS.

Fractures of the Maxillæ.... 528
Dangers of Hypnotism.... 531
That Barbaric "Key".... 533
Importance of Dentistry to the Physician .... 535

PROCEEDINGS.

The National Association of Dental Examiners .... 536
Constitution of the National Association of Dental Examiners. .... 545
National Association of Dental Faculties.... 548

SELECTIONS.

Facial and Thoracic Deformities Incident to Obstruction by Adenoid Hypertrophy in the Naso-Pharynx.... 551
World-Wide Brotherhood.... 553
Some Notes on the Nails.... 554
Disinfecting Means and Methods.... 557
The Action of Microbial Products on Microbes and on the Organism.... 559
Removal of Breast During Hypnotic Sleep.... 561
For Trigeminal Neuralgia.... 562
New Method for Vulcanizing Rubber Plates.... 562
Remedy for La Grippe.... 563
A Case of Congenital Lockjaw.... 564
Antiseptics among the Ancient Greeks.... 565
An Infant Prodigy.... 566
Mania Following Ether.... 567
Effects of Heat and Cold—How to Kill Bacteria.... 567
Evolution in Dentistry.... 568

EDITORIAL.

New Arrangement—Editorially.... 572
Prof. W. D. Miller.—A Reception.... 572
The Metric System .... 574
Are Dentists Honest?.... 575

